# Intravesical urachal cyst masquerading as a bladder malignancy: a case report

**DOI:** 10.1186/s13256-023-04110-w

**Published:** 2023-08-24

**Authors:** Zahra Alyusuf, Ali Hassan, Reem Maki, Wafa Hasan, Roaa Alhamar

**Affiliations:** Radiology Department, Salmaniya Medical Complex, Manama, Bahrain

**Keywords:** Bladder malignancy, Congenital urachal anomaly, Intravesical urachal cyst, Magnetic resonance imaging, Rhabdomyosarcoma, Ultrasound

## Abstract

**Background:**

Urinary bladder masses in children are extremely rare. Certain benign conditions (e.g., ureterocele) can mimic malignant bladder masses. In this report, we present a unique case of a urachal cyst masquerading as a bladder malignancy. Unlike the typical location of urachal cysts along the course of the urachal tract, the cyst in this case was unexpectedly situated within the urinary bladder, leading to diagnostic difficulties.

**Case presentation:**

A 2-year-old Bahraini boy presented with hematuria and dysuria for 2 weeks. There was no history of fever, abdominal pain, or vomiting. Physical examination yielded normal findings. Urinalysis showed numerous red blood cells and revealed positive results for nitrites and leukocyte esterase. Abdominal ultrasound showed a well-defined soft tissue lesion with internal vascularity located at the apex of the urinary bladder. Subsequently, magnetic resonance imaging demonstrated a thick-walled cystic structure arising from the anterosuperior wall of the bladder and protruding into its lumen. The patient underwent complete excision of the bladder lesion for the presumed diagnosis of rhabdomyosarcoma. Histopathological examination showed a fluid-filled space lined by stratified squamous epithelium with areas of intestinal metaplasia, revealing an unexpected diagnosis of a urachal cyst. The patient was discharged with complete resolution of symptoms.

**Conclusions:**

Intravesical urachal cysts are a rare type of congenital urachal anomaly that may simulate a bladder malignancy, particularly if associated with infection. This case emphasizes the importance of considering urachal cysts in the differential diagnosis of bladder masses, especially in children, and specifically when the lesion is midline in the anterosuperior wall of the bladder.

## Background

Unlike in adults, urinary bladder masses in children are extremely rare and have distinct histologic types. Except for rhabdomyosarcoma, which is the most common pediatric bladder malignancy, most bladder masses are benign. These include fibroepithelial polyps, inflammatory myofibroblastic tumors, urothelial papilloma, neurofibroma, and leiomyoma [1]. Children with bladder masses typically present with lower urinary tract symptoms, such as dysuria, frequency, and hematuria. Ultrasound remains the first-line imaging investigation in the evaluation of these patients.

Certain potential pitfalls may lead to erroneous reporting of bladder masses on ultrasound [2]. For example, anatomical defects (e.g., ureteroele), pelvic masses (e.g., ovarian cysts), or mobile materials within the bladder (e.g., calculi) may be misinterpreted as masses arising from the urinary bladder [2, 3]. Additionally, non-neoplastic inflammatory conditions such as eosinophilic cystitis, may simulate bladder masses [4]. Here, we report a rare case of an intravesical urachal cyst that was clinically and radiologically masquerading as a bladder malignancy. Urachal cysts typically have an extravesical location along the course of the urachal tract. The intravesical location of urachal cysts is a rare and recently recognized type of congenital urachal anomaly [5, 6].

## Case presentation

A 2-year-old Bahraini boy was brought to the emergency department by his mother due to the presence of blood in his urine and diaper for two weeks. The child also complained of painful urination and foul-smelling urine. The patient had no history of fever, bruising, abdominal pain, or vomiting, and there was no reported trauma or upper respiratory tract infection. The child had a medical history of eczema and delayed speech. The family history was unremarkable.

On physical examination, the child appeared active and well-nourished, with vital signs within normal limits. Abdominal examination revealed the abdomen to be soft, non-tender, and without palpable masses, with normal bowel sounds in all quadrants. The umbilicus appeared normal, with no visible abnormalities or signs of discharge. Examination of other bodily systems also revealed normal findings.

Urine analysis showed > 100 red blood cells per high-power field, 50 white blood cells per high-power field, and positive results for nitrite and leukocyte esterase. Other laboratory investigations, including hematological and biochemical parameters, showed no abnormalities.

**Fig. 1:**
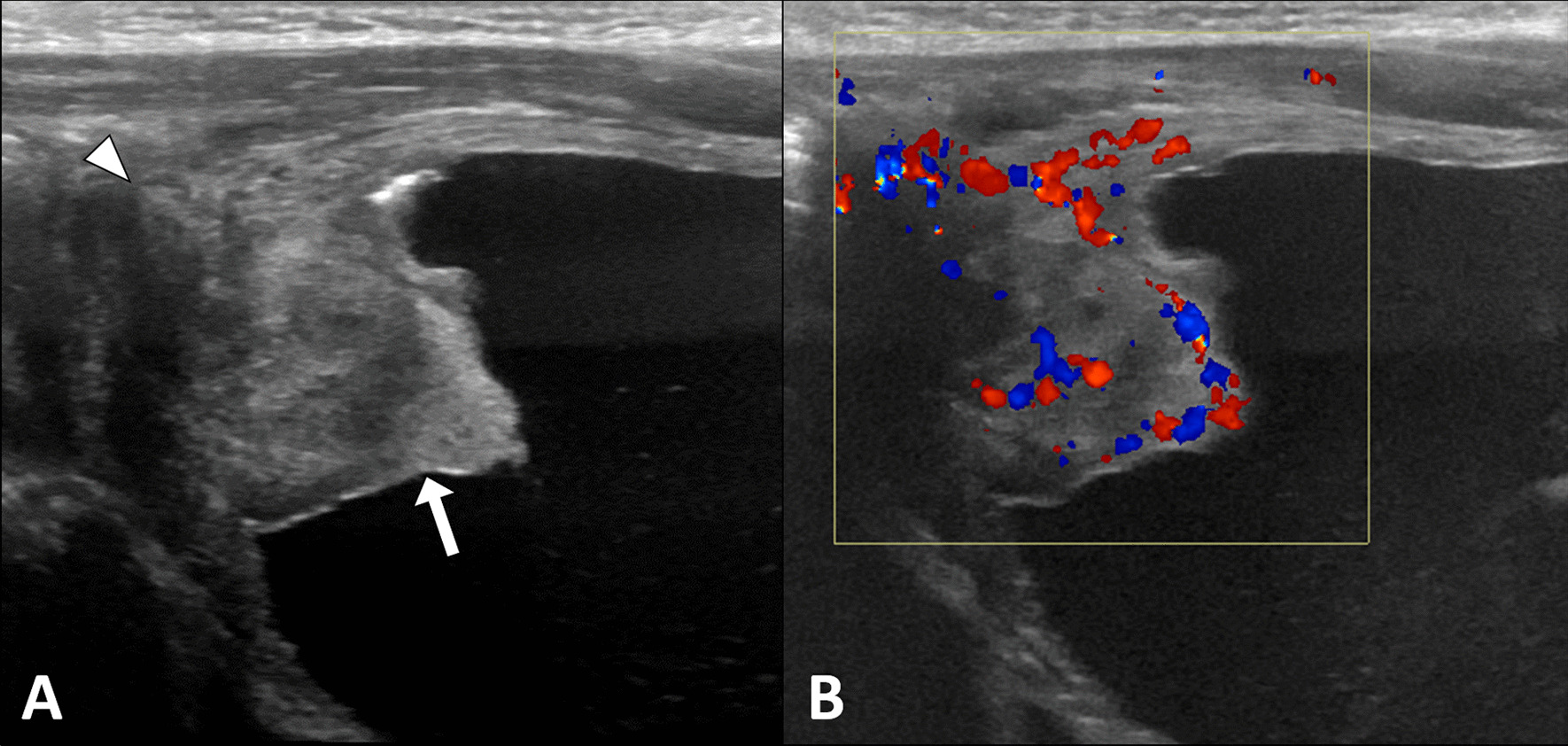
Ultrasound image (**A**) of the urinary bladder shows a soft tissue lesion (arrow) with an area of suspicious extravesical extension (arrowhead). The color Doppler image (**B**) shows internal vascularity within the lesion

**Fig. 2:**
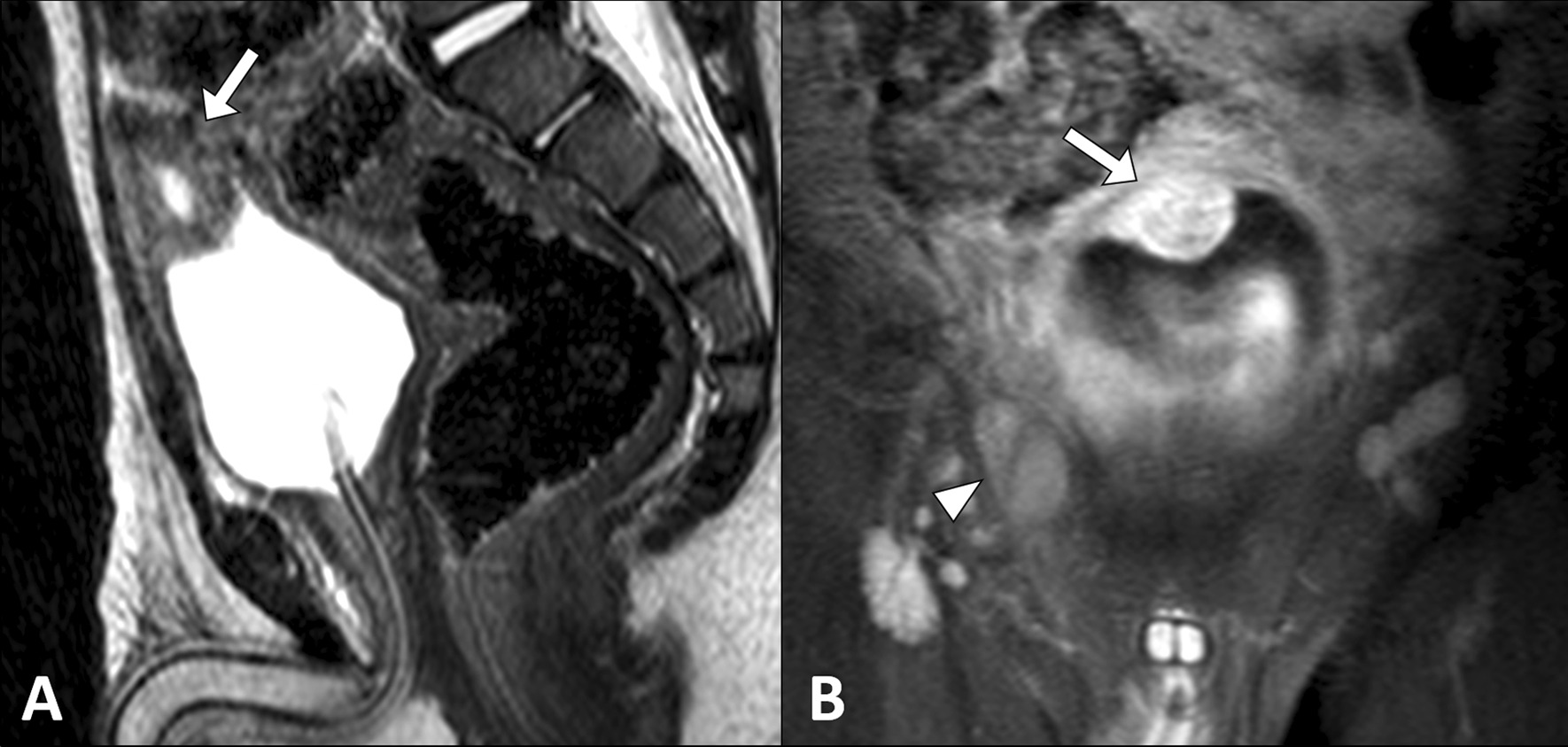
T2-weighted magnetic resonance image (**A**) shows a cystic lesion (arrows) arising from the anterosuperior wall of the urinary bladder and protruding into its lumen. Coronal, post-contrast, fat-suppressed, T1-weighted image (**B**) shows heterogeneous enhancement of the lesion, with an incidental finding of retractile right testis in the inguinal canal (arrowhead)

To rule out any underlying congenital urinary tract abnormalities as the cause of the urinary tract infection, an abdominal ultrasound examination was performed. The scan revealed a well-defined, lobulated, soft tissue lesion arising from the apex of the urinary bladder with internal vascularity on color Doppler. There was a focal irregularity of the bladder wall at the site of the lesion, raising the possibility of an extravesical extension. Low-level mobile echoes were also observed within the lumen of the bladder (Fig. 1). Both kidneys had normal echogenicity and morphology with no evidence of hydronephrosis, nephrolithiasis, or focal masses. Pelvic magnetic resonance imaging (MRI) demonstrated a thick-walled cystic lesion arising from the anterosuperior wall of the bladder and protruding into its lumen. The lesion measured 2.0 cm × 1.3 cm × 2.0 cm (anteroposterior × transverse × craniocaudal). It was isointense to muscles on T1-weighted images, hyperintense on T2-weighted images, and exhibited heterogeneous post-contrast enhancement and restricted diffusion. Mild thickening and post-contrast enhancement of the bladder wall were observed at the site of the lesion. No other bladder wall lesions were identified, and no regional lymphadenopathy was noted. The right testis was seen in the inguinal canal (Fig. 2).

After a thorough evaluation, the multidisciplinary team determined that the best course of action was laparotomy with segmental cystectomy for the suspected rhabdomyosarcoma. The decision was based on several factors, including the symptomatic nature of the lesion, highly suspicious initial clinical and radiological findings, and the technical difficulties of performing a cystoscopic biopsy in pediatric patients, where specialized equipment required for the procedure was not readily available, and delaying management was not advisable. The team also considered the potential for perforation and seeding of malignant cells into the peritoneal cavity due to the location of the lesion.

**Fig. 3:**
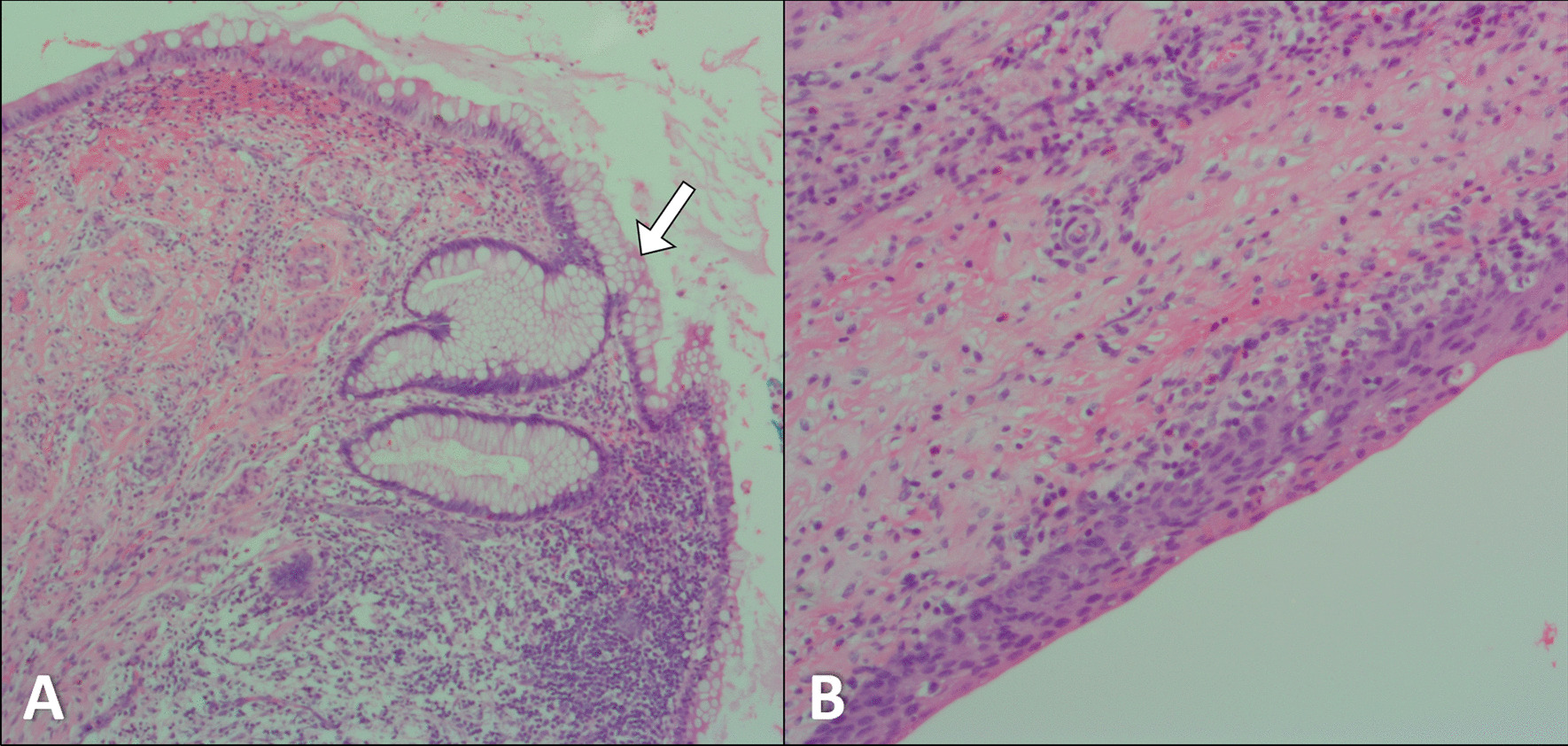
Histopathological images (hematoxylin and eosin stain) in low-power (**A**) and high-power (**B**) views show a fluid-filled space lined by a stratified squamous epithelium with focal areas of intestinal metaplasia (arrow)

The patient underwent laparotomy and complete excission of the lesion. A gross examination revealed a firm mass with frond-like papillary projections. Subsequent histopathological examination revealed a fluid-filled space lined by stratified squamous epithelium with areas of intestinal metaplasia, without any evidence of atypia or malignancy. The immunohistochemistry results were negative for ALK1, MyoD1, and cytokeratin AE1/AE3. These unexpected findings led to the diagnosis of a benign urachal cyst (Fig. 3).

Postoperatively, the medical team closely monitored vital signs and hydration status. A urinary catheter was kept in place for 1 day to monitor for bleeding and ensure proper bladder drainage. A strict regimen of hygiene practices was implemented to prevent infection, and pain management was closely monitored to ensure the patient's comfort. The patient was discharged on the second postoperative day with normal urine analysis results. At the 1-month follow-up visit, the mother reported no active complaints, and there was no recurrence of the initial symptoms.

## Discussion and conclusions

We present a rare case of intravesical urachal cyst masquerading as a bladder malignancy. The urachus is a tubular embryonic structure that connects the fetal urinary bladder to the allantois. Normally, the urachus is obliterated at birth and forms a fibromuscular structure known as the median umbilical ligament [7]. However, when the urachus fails to involute, it leads to a spectrum of congenital anomalies, including four types of urachal anomalies: patent urachus, umbilical urachal sinus, vesicourachal diverticulum, and urachal cyst [8]. In 2013, Metwalli et al. [6] expanded this spectrum of anomalies by describing a distinct type of urachal cysts that protrudes from the anterosuperior wall of the bladder into the bladder lumen, which they termed intravesical urachal cysts.

Intravesical urachal cysts are a recently recognized congenital urachal anomaly [5, 6]. Yagishita et al. [5] reported the histopathological findings of an incidental intravesical urachal cyst in an adult patient with four additional similar cases from the Japanese medical literature, but the imaging features of such cases were not described. Microscopically, the cyst had a thin wall with a cuboidal epithelial lining and underlying fibrous and muscular tissues [5]. Metwalli et al. [6] reported five cases of intravesical urachal cysts in children with a clear description of their sonographic findings. However, the diagnosis was not confirmed by histopathology in 4 of these cases. On ultrasound, an intravesical urachal cyst appeared as a thin-walled ovoid midline structure arising from the anterosuperior part of the bladder and could have an acute or obtuse angle with the bladder wall [6]. In the present case, the urachal cyst did not demonstrate the classic sonographic findings and exhibited heterogeneous echogenicity, making it indistinguishable from bladder malignancy. Such atypical findings of urachal cysts may occur in the setting of associated infection [3]. MRI can be performed to confirm the diagnosis and provide additional information on the lesion with respect to its exact location and anatomical relationships. On MRI, the urachal cyst appears as a thin-walled midline structure located along the course of the urachal tract between the bladder and the umbilicus. It typically has a low signal intensity on T1-weighted images, high signal intensity on T2-weighted images, and no enhancement on post-contrast images [3]. However, in the present case, the intravesical urachal cyst had a thick wall and demonstrated post-contrast enhancement and restricted diffusion. The scan also revealed an incidental finding of a retractile right testis in the inguinal canal. Certain genitourinary conditions may be associated with urachal anomalies, including vesicoureteral reflux, hypospadias, anal stenosis, and cryptorchidism [9, 10].

Congenital urachal anomalies are relatively uncommon [11, 12]. Given the widespread use of imaging examinations, urachal anomalies can be found incidentally [11]. Urachal anomalies may present clinically with urinary or abdominal symptoms if they attain a large size or develop complications, including infection and malignancy [8].

Rhabdomyosarcoma was initially suspected in the present case before the histopathological diagnosis confirmed it as a urachal cyst. Rhabdomyosarcoma typically presents within the first 5 years of life and tends to affect males more frequently [13]. Its characteristic location in the bladder trigone and bladder neck contrasts with intravesical urachal cysts [3], which are characterized by their midline position in the anterosuperior wall of the bladder [5, 6], making this distinction crucial for accurate diagnosis.

On ultrasound, rhabdomyosarcoma appears as a predominantly solid, round or lobulated mass with internal flow on color Doppler [3]. On MRI, rhabdomyosarcoma exhibits isointensity or hyperintensity to muscles on T1-weighted images, and hyperintensity on T2-weighted images due to necrosis, with postcontrast enhancement varying depending on tumor size [3]. The clinical and imaging characteristics in the present case raised suspicion for rhabdomyosarcoma, underscoring the importance of endoscopic biopsy and histopathological examination of suspicious bladder masses before considering surgical interventions. Histopathologically, rhabdomyosarcoma encompasses several subtypes, with the embryonal subtype being the most common, accounting for over 90% of cases, including botryoid and variant spindle cell forms. It is characterized by small, dark, spindle-shaped, or round cells with minimal cytoplasm, intermixed with a variable number of cells resembling rhabdomyoplasts [3, 14].

The management of congenital urachal anomalies is controversial. Some authors advocate using a conservative approach in asymptomatic or even mildly symptomatic patients, as it became increasingly recognized that spontaneous regression of urachal remnant may occur [15, 16]. Traditionally, the management approach has been surgical excision even in incidental cases. However, the risk of malignant transformation was found to be extremely low [11]. It is estimated that more than 5,700 cases of urachal anomalies must be excised to prevent a single case of urachal adenocarcinoma [11]. Surgical excision is clearly indicated for large or suspicious urachal lesions, regardless of symptoms [16]. Cystoscopy-assisted laparoscopy is a feasible minimally invasive approach and has the advantages of rapid recovery and reduced blood loss [17].

Intravesical urachal cysts are a rare, yet important, type of congenital urachal anomaly. In unusual instances, particularly if associated with infection, urachal cysts may have atypical radiologic features that can be indistinguishable from bladder malignancy. It is important to always consider the differential diagnosis of urachal cysts in patients with a focal midline mass related to the anterosuperior wall of the urinary bladder. An accurate diagnosis of this condition is crucial to provide reassurance to patients and obviate unnecessary radical surgical procedures.

## Data Availability

Not applicable.
